# Current Status of Drug Treatment of Cholangiocarcinoma—Updated Progress and Critical Limitations

**DOI:** 10.3390/ph19040554

**Published:** 2026-03-31

**Authors:** Jennifer Cillis, Courtney Chen, Supriya Deshpande, Yuman Fong, Shyambabu Chaurasiya

**Affiliations:** 1 Department of Surgery, City of Hope, Duarte, CA 91010, USAyfong@coh.org (Y.F.); 2 Department of Surgery, Rutgers Robert Wood Johnson Medical Center, New Brunswick, NJ 08901, USA; 3 Department of Surgery, Cedars Sinai Medical Center, Los Angeles, CA 90048, USA

**Keywords:** bispecific T cell engagers, chemotherapy, chimeric antigen receptor (CAR) T cells, cholangiocarcinoma, fibroblast growth factor receptor (FGFR), immunotherapy, inhibitors, oncolytic viruses (OVs), targeted therapy

## Abstract

Cholangiocarcinoma (CCA) is a highly lethal, heterogeneous malignancy arising from the biliary tract. Although the prevalence of CCA is relatively low, its incidence has increased in the last few decades, and the overall prognosis is poor. Surgical resection remains the most efficacious treatment modality for CCA. However, due to its aggressive nature and often asymptomatic presentation, most patients are first diagnosed with advanced disease, precluding them from curative intervention. Moreover, due to its heterogeneity at the molecular, genomic, and epigenetic levels, drug treatment of CCA remains challenging. In this review, we discuss the current standard drug treatment approaches, recent breakthroughs, and promising new therapeutics for CCA. We summarize key clinical data for the standard first-line chemotherapy regimen and its efficacy and resistance mechanisms, along with more recent studies supporting or proposing second-line treatments. We highlight landmark clinical trials, including ABC-02, which established gemcitabine-cisplatin (GC) as the first-line regimen against biliary cancers. Additionally, we discuss recent findings on the susceptibility of CCA against targeted therapies and other immunologic molecules, including results from the KEYNOTE-966 and TOPAZ-1 clinical trials. Finally, we critically analyze new therapeutics in the preclinical and clinical space, such as CAR-T cells and oncolytic viruses that may be effective against CCA.

## 1. Introduction

Cholangiocarcinoma (CCA) is the most common primary malignancy of the biliary tree. CCA comprises approximately 15% of all primary liver tumors and is the second-most-common primary hepatic malignancy after hepatocellular carcinoma (HCC) [1]. CCA is heterogenous in nature and classified as either intrahepatic (iCCA; tumor proximal to second-order bile ducts) or extrahepatic (eCCA; the second-order bile ducts serve as the line of demarcation) [2]. Extrahepatic CCA is further separated, based on its location relative to the insertion of the cystic duct, into perihilar (pCCA; tumor between second-order bile ducts and cystic duct-bile duct junction) and distal (dCCA; tumor between cystic duct-bile duct junction and the ampulla of Vater) [2,3]. Each subtype has distinct mutations, clinical phenotypes, and therapeutic strategies.

### 1.1. Epidemiology

*Risk factors*: Multiple risk factors are associated with CCA, including cirrhosis, hepatitis B (HBV), and hepatitis C (HCV) [4,5]. Congenital conditions, such as Caroli disease and choledochal cysts are also associated with CCA. Additional predisposing factors include biliary duct cysts and hepatolithiasis [5,6]. Several environmental factors are suspected to be related to CCA development, including nitrosamide-contaminated food, dioxins, and vinyl chlorides [7]. Liver fluke (*Opisthorchis viverrini* and *Clonorchis sinesis*) infection is a common risk factor in East Asia, while primary sclerosing cholangitis is commonly associated with CCA in Western countries [7,8]. Several studies have shown an association of iCCA with metabolic syndrome and non-alcoholic liver disease, which is significant due to the increasing incidence of obesity in Western countries [5,7,8,9].

*Molecular pathogenesis*: New innovations in gene sequencing and molecular reporting have increased our understanding of genetic mutations associated with CCA. The most commonly mutated genes (mutation frequencies 10–26%) in CCA include tumor protein 53 (*TP53*), AT-rich interaction domain 1A (*ARID1A*), Kirsten rat sarcoma virus (*KRAS*), SMAD family member 4 (*SMAD4*), BRCA1-associated protein 1 (*BAP1*), and adenomatous polyposis coli (*APC*), followed by several other genes with lower mutation frequencies of 1–6% [10,11,12,13,14,15,16,17,18]. Many prominent cellular pathways have been found to be dysregulated in CCA cases, including the inflammation-related interleukin 6 (IL-6)/Janus kinase (JAK)/signal transducer and activator of transcription 3 (STAT3) pathway [19], the epidermal growth factor receptor (EGFR)/mitogen-activated protein kinase (MAPK)/extracellular signal-regulated kinase (ERK) pathway, and hepatocyte growth factor/AKT serine/threonine kinase (AKT)/ERK signaling [20,21].

Furthermore, comprehensive whole-exome and transcriptome studies have been performed in large patient cohorts, which have revealed potentially targetable genetic driver alterations in ~40% of CCA patients [12]. However, the frequencies of driver gene mutations differ between CCA subtypes. For example, mutations in isocitrate dehydrogenase 1/2 (*IDH1/2*), fibroblast growth factor receptor 1/2/3 (*FGFR1/2/3*), ephrin type-A receptor 2 (*EPHA2*), and *BAP1* are found primarily in iCCA cases, while *ARID1B*, E74-like ETS transcription factor 3 (*ELF3*), polybromo 1 (*PBRM1*), protein kinase cAMP-activated catalytic subunit alpha (*PRKACA*), and protein kinase cAMP-activated catalytic subunit beta (*PRKACB*) mutations are discovered mostly in eCCA cases [12]. Identifying these genetic abnormalities furthers our understanding of the distinct CCA subtypes and provides potential candidates for targeted therapies.

The progression to CCA is likely multifactorial due to predisposing risk factors in combination with aberrant molecular pathways. For example, infection with the liver fluke *Opisthorchis viverrine* results in chronic inflammation and damage to the bile ducts, characterized by infiltration of neutrophils and mononuclear cells [22]. Proteins and other metabolites released by the *Opisthorchis viverrine* worms activate Toll-like receptor 4 (TLR-4) and induce expression of pro-inflammatory cytokines, including IL-8 and IL-6 [23]. Gene polymorphisms and mutations can alter cytokine expression and affect inflammatory processes, including activation of the IL-6/JAK/STAT3 pathway. People infected with *Opisthorchis viverrine* who also have dysregulated pro-inflammatory cytokine production are more susceptible to developing bile duct fibrosis and CCA [24].

*Incidence*: The global incidence of CCA varies, reflecting the regional prevalence of risk factors [9,25]. Although CCA is a rare cancer (<6 cases per 100,000 people) in most countries, some—including South Korea, Thailand, and regions of China—have significantly higher incidence rates [9,26]. Together, pCCA (50–50%) and dCCA (20–30%) comprise approximately 80% of CCA diagnoses in the U.S., while iCCA accounts for the remaining 20% of cases [27,28]. Globally, the incidence and mortality of iCCA have increased, whereas the incidence of eCCA has remained relatively unchanged [25,29]. In the U.S., iCCA incidence has increased over both 10- and 40-year periods, while the incidence of eCCA has remained stable [29]. Additionally, CCA mortality rates have increased by almost 40% in recent years—from 2.2 per 100,000 people to 3.0 per 100,000—primarily due to the increased incidence of iCCA cases [30,31].

However, interpretation of rising iCCA rates is complicated because previous versions of the International Classification of Diseases (ICD) did not include a separate code for pCCA, and previous versions of ICD-Oncology (ICD-O) cross-referenced pCCA to iCCA. Not having a unique code for pCCA may have led to widespread inaccuracies, especially with mislabeling pCCA as iCCA [1,32]. In a study in the UK published in 2021, retrospective review of 625 hepatobiliary cancers from three independent centers found that only 43% of CCAs were truly pCCA, while 92% of those that were actually pCCA were incorrectly coded as iCCA [33]. Thus, the mislabeling of pCCA as iCCA may be influencing the apparent rise in iCCA rates. The next iterations of both the ICD and ICD-O (ICD-11 and ICD-O-4) will have separate codes to record iCCA, pCCA, and dCCA, facilitating more accurate epidemiology moving forward [34].

### 1.2. Diagnosis

CCA is difficult to diagnose due to its silent clinical character. Due to restricted anatomic access, most diagnostic methods have low sensitivity for identifying these tumors. Most patients with CCA develop symptoms, such as abdominal pain or jaundice, only at an advanced stage of disease, resulting in a poor prognosis with a median survival of 24 months after diagnosis [35,36] Cancer antigen 19-9 (CA 19-9) is the primary serum biomarker used in the diagnosis of CCA, with CA 19-9 levels > 1000 U/mL associated with metastatic disease [37,38,39].

iCCA is usually detected as a hepatic mass lesion, often via computed tomography (CT) or magnetic resonance imaging (MRI) modalities. CT and MRI have comparable performance in the detection of primary and satellite iCCA lesions, but CT imaging is superior for the detection of vascular enhancement, which is necessary for assessment of resectability [40]. A combination of CT and MRI with magnetic resonance cholangiopancreatography (MRCP) imaging is utilized for the detection of pCCA and dCCA. MRI-MRCP has higher diagnostic accuracy for the detection of biliary neoplastic invasion, while CT has superior assessment of vascular involvement [41,42]. Additionally, endoscopic retrograde cholangiopancreatography (ERCP) plays an important role by facilitating the detection of malignant biliary strictures and acquiring brushing samples for cytological and genetic evaluation [43].

Cytological diagnosis of CCA is not always possible when tumors are located in inaccessible areas of the biliary tree. However, there are several cytological techniques that have utility in diagnosing CCA when biopsy specimen can be collected. An optimized fluorescent in situ hybridization (FISH) probe set has been developed to detect pancreaticobiliary malignancies, including CCA, with a sensitivity of 93% and specificity of 100% [44]. While FISH-based assays can detect gene fusions and amplifications with high specificity, they are limited by the number of targets that can be assessed simultaneously [45]. Next-generation sequencing (NGS) for known or candidate oncogenic targets can improve the diagnostic function of conventional biliary cytology. In 33 patients with malignant-appearing pancreaticobiliary strictures, NGS combined with cytology had a sensitivity of 85% in detecting high-risk neoplasia or malignancy, compared with 67% for cytology alone [46]. Some drawbacks of NSG include its high cost, required specialized equipment, and the need for high cell counts from tissues for accurate analysis [45].

Furthermore, molecular advances in techniques utilizing circulating tumor DNA (ctDNA) and cell-free DNA (cfDNA) have the potential to be used for detection and disease monitoring in CCA cases. Liquid biopsies can be performed to detect ctDNA, cfDNA, or other tumor biomarkers from blood samples, followed by polymerase chain reactions (PCR) or NGS to identify gene mutations [47]. This is less invasive than traditional tissue biopsy and enables genomic profiling of tumors with limited accessibility [48]. Recent studies have assessed concordance between ctDNA and tissue genomic profiling, including one performed in a cohort of patients with advanced biliary tract cancer. The concordance between patient ctDNA and tumor tissue mutations demonstrated a sensitivity of 84.8% and a positive predictive value of 79.4% [48]. However, developing these liquid biopsy assays can be time-consuming and expensive, which may limit their application in clinical practice. Additionally, low concentrations of biomarkers compared to other blood components makes isolating and analyzing ctDNA and cfDNA challenging [47].

### 1.3. Standard-of-Care Surgical Treatment

Surgical resection is currently the only potentially curative treatment for all three CCA subtypes. The surgical approach varies between the subtypes based on their underlying anatomical differences. For iCCA cases, this includes a hepatic lobectomy with intent to attain a margin-negative (R_0_) resection [49]. Staging laparoscopy is recommended to detect any occult metastatic disease not apparent on staging imaging, especially in patients with high-risk features [50]. Poor post-operative disease-free survival (DFS) is associated with large tumor size, multiple liver lesions, regional lymph node involvement, and positive resection margins [51].

Liver transplantation has become a consideration for certain subsets of iCCA patients, particularly those with small (≤2 cm) unresectable tumors and concomitant cirrhosis [1]. Retrospective studies have also identified patients who underwent liver transplantation with incidental iCCA or mixed HCC/CCA with tumor diameters between 2 cm and 5 cm [52]. Recurrence-free survival (RFS) was 74% at 5 years, and transplant outcomes were similar to patients who had tumors < 2 cm [53,54]. This suggests that cirrhotic patients with iCCA tumors up to 5 cm may have acceptable outcomes after liver transplant. Additional retrospective studies reported that patients with locally advanced iCCA who showed pre-transplant disease stability after neoadjuvant chemotherapy benefited from liver transplantation, with 83% 5-year overall survival (OS) rates [55]. The criteria for liver transplantation in iCCA cases are evolving, with several prospective clinical trials currently ongoing.

For pCCA, resection with curative intent often involves lobectomy with bile duct resection, regional lymphadenectomy, and Roux-en-Y (RnY) hepaticojejunostomy [56]. Surgical developments, including extended lobectomy and vascular reconstruction, have enabled the resection of tumors that were previously considered unresectable [57,58,59,60]. Liver transplantation is another option, with several studies illustrating that liver transplantation has better overall survival when compared to resection with curative intent [61,62]. Surgical resection of dCCA typically involves a pancreaticoduodenectomy (Whipple procedure) with removal of the gallbladder and bile duct, the head of the pancreas, and the first part of the duodenum [63].

While the singular curative treatment for CCA is surgery, the often late-stage presentation of CCA precludes up to 75% of patients from being eligible for surgery [36,64]. Even after resection, post-operative relapse of CCA is common. Therefore, drug-based treatment of CCA remains mainstay, with numerous clinical trials ongoing to optimize potential treatment regimen. Additionally, targeted therapies, including monoclonal checkpoint inhibitors, oncolytic viruses (OVs), and chimeric antigen receptor (CAR) T cells, are emerging options for CCA treatment.

## 2. Chemotherapy

### 2.1. First-Line Regimen—Gemcitabine + Platinum

Given the late presentation of CCA, several clinical trials have aimed to identify a superior chemotherapy regimen for these patients. In numerous clinical trials, CCA has been grouped with gallbladder cancer under the umbrella terms “biliary tract adenocarcinoma” or “biliary tract cancer.” One of the earlier trials utilized gemcitabine-oxaliplatin (GEMOX) as a combination treatment for advanced biliary tract adenocarcinoma (ABTA), based on its prior use in pancreatic adenocarcinoma. In this phase II study named GERCOR, 56 patients were treated with GEMOX. Patients were divided into group A (*n* = 33) or group B (*n* = 23) based on various parameters. Group A patients had no history of prior chemotherapy or radiotherapy, a performance status of 0–2, hematological parameters within normal limits, and adequate renal and liver function. Group B patients had prior chemotherapy or radiotherapy treatments and any parameters that did not qualify them for group A, including poor performance status or elevated bilirubin levels. In group A, 11 patients had a partial response, eight had stable disease, and 12 patients had disease progression for a response rate of 35.5%. Median progression-free survival (PFS) and OS were 5.7 and 15.4 months, respectively. In group B, one patient had a complete response, four had a partial response, and 11 had disease progression, giving a response rate of 22%. Median PFS and OS were 3.9 and 7.6 months. These results demonstrated that GEMOX was safe and well tolerated, had a higher response rate, and improved survival in patients with better overall prognostic indicators. GEMOX had some efficacy in group B, which comprised patients who had received previous chemotherapy and had an overall poorer prognosis. This study established the efficacy of the GEMOX regimen in ABTA [65].

Gemcitabine plus cisplatin (GC) combination therapy has become standardized treatment for several cancer types, and it has been effective in the treatment of BTC. A multicenter, randomized phase II conducted in Japan directly compared GC combination therapy to gemcitabine treatment alone in chemotherapy-naïve patients, with locally advanced or metastatic BTC. Eighty-three patients were treated with GC (*n* = 41) or gemcitabine alone (*n* = 42). There were no complete tumor responses, but the disease control rate was 68.3% in the GC arm vs. 50% in the gemcitabine arm. The 1-year survival rate was 39% vs. 31%, with a response rate of 19.5% vs. 11.9%, in favor of the combination therapy. The median survival time was 11.2 months vs. 7.7 months, and median PFS was 5.8 months vs. 3.7 months, also in favor of the GC arm. The most common grade 3 or higher adverse events in the GC arm were neutropenia, thrombocytopenia, and hemoglobin decrease, which were all manageable and the patients recovered [66]. These results demonstrated the superiority of GC combination therapy in direct comparison with gemcitabine treatment alone, with minimal concerns for treatment-limiting toxicity (Table 1).

Further studies were performed to directly compare GC chemotherapy to gemcitabine alone, including ABC-02—an extension of the ABC-01 trial—a randomized, controlled phase III trial in the United Kingdom (NCT00262769). These 410 patients with locally advanced or metastatic CCA, gallbladder cancer, or ampullary cancer were randomly assigned to receive GC (*n* = 204) or gemcitabine alone (*n* = 202). The median follow-up was 8.2 months, with median OS of 11.7 months among the patients in the GC group, compared to 8.1 months in the gemcitabine group. The median PFS was 8 months in the GC group and 5 months in the gemcitabine group. Additionally, the rate of tumor control among patients in the GC group significantly increased to 81.4% compared to 71.8% in the gemcitabine group. Adverse events were similar in the two groups, except for more instances of neutropenia in the GC group. However, the number of neutropenia-associated infections was similar between the two groups. This clinical trial demonstrated that GC offers a significant survival advantage over gemcitabine monotherapy and is a viable treatment option for patients with advanced biliary cancers (ABCs) [67].

Pooled analysis of chemotherapy trials from 1985 to 2006 was performed to compare response rate, tumor control rate, and OS for several chemotherapy drugs as monotherapies and in combination. Chemotherapy compounds included fluoropyrimidines, gemcitabine, platinum compounds, and taxanes. These analyses showed significant superiority of dual therapy compared with monotherapy in relation to response rate and tumor control rate. Also, treatment with gemcitabine and platinum compounds consistently resulted in highly superior response rates and tumor control rates compared with gemcitabine-free or platinum-free combinations. The addition of platinum compounds increased the response rate of both gemcitabine and fluoropyrimidines. The increase in response rate from adding platinum compounds to gemcitabine was twice as high as that observed when platinum was added to fluoropyrimidines (17.0 vs. 8.7%). This large-scale study demonstrated that gemcitabine combined with platinum compounds should be the provisional standard chemotherapy regimen for ABCs [76].

### 2.2. Second-Line Regimen—FOLFOX or FOLFIRI

Some patients progress after first-line GC, which necessitates a second-line regimen for those who can tolerate further chemotherapy. Three types of drug classes—gemcitabine, fluoropyrimidines, and platinum-based drugs—have shown activity against BTC. Switching to a fluoropyrimidine-based treatment after progression on first-line gemcitabine-based chemotherapy has been deemed appropriate for other types of cancer, including pancreatic cancer [69]. Therefore, one treatment that was anticipated to be effective against CCA was fluorouracil plus a platinum doublet. The FOLFOX regimen comprises leucovorin, fluorouracil, and oxaliplatin. The ABC-06 study, a randomized, controlled phase III prospective trial, aimed to study whether patients with ABC benefit from the addition of second-line FOLFOX after progression of their disease on GC. One hundred and sixty-two patients were enrolled, with 81 patients randomly assigned to each group. OS in the group that received FOLFOX was 6.2 months compared to 5.3 months in the control group. The OS rate in the FOLFOX group was 50.6% at 6 months and 25.9% at 12 months, compared to a 35.5% survival rate at 6 months, and an 11.4% survival rate at 12 months, in the control group [69]. The most frequently reported grade 3–5 FOLFOX-related adverse events were neutropenia, fatigue, and infection. This study exhibited that FOLFOX improved median OS in patients with ABC after progression on GC, highlighting the potential benefit of second-line treatment in patients.

Other groups have examined additional possible second-line treatment regimens. One study retrospectively reviewed patients with ABC who received GEMOX-based chemotherapy followed by 5-fluorouracil-irinotecan (FOLFIRI)-based chemotherapy to examine this possible sequential treatment strategy. Fifty-two patients were examined, 21 of which had iCCA, 14 had eCCA, and 17 had gallbladder cancer. After a median follow-up of 36 months, 90% of patients had completed the treatment strategy. The OS for the sequential GEMOX-FOLFIRI chemotherapy was 21.9 months [77]. This study demonstrated that the sequence of GEMOX-FOLFIRI is a potential treatment strategy for patients with recurrent or ABC.

Additional studies retrospectively identified patients with ABC who were treated with the FOLFIRI regimen. Ninety-eight patients were included, with 74 having metastatic disease and 24 having locally advanced disease at the time of treatment. FOLFIRI was used as 1st-, 2nd-, 3rd-, or 4th-line treatment in 8, 50, 36, and 4 patients, respectively. In the entire cohort, the median PFS was 2.4 months, and OS was 6.6 months. Median PFS for patients treated with FOLFIRI in 1st-, 2nd-, 3rd-, or 4th-line treatment were 3.1, 2.5, 2.3, and 1.5 months, respectively. There was no statistically significant difference in median PFS for patients with locally advanced disease compared to those with distant metastases at time of treatment. Given the lack of standard therapy for patients who experience disease progression on first-line chemotherapy, FOLFIRI may be a treatment option with its modest efficacy [78].

A single institution retrospective study was performed to evaluate PFS for patients who underwent various second-line chemotherapy regimens. Fifty-six patients were included, and the majority had advanced iCCA. Eighty percent of these patients had previously received first-line gemcitabine-based therapy. The second-line therapies patients received included gemcitabine plus platinum (19.6% of patients), gemcitabine plus fluoropyrimidine (28.6%), fluoropyrimidine combination (37.5%), and other combinations (14.3%). Median PFS was 2.7 months with a median OS of 13.8 months, and a 50% disease control rate. There was no significant difference in survival between these four treatment groups [79]. Prospective randomized clinical trials like the ABC-06 study have demonstrated the potential clinical benefit of FOLFOX as a second-line chemotherapy, with several retrospective studies demonstrating that other regimens such as FOLFIRI may have survival benefits. Additional prospective randomized clinical trials examining various second-line chemotherapy regimens, especially in direct comparison to each other, would further explore the therapeutic options for patients who progressed on first-line therapy.

### 2.3. Adjuvant Chemotherapy—Capecitabine

As previously discussed, surgical resection is the only curative treatment for CCA. Among patients who undergo surgical resection, the postoperative median OS is between 18–30 months. However, those with positive lymph nodes and positive resection margins have a worse prognosis [72]. Due to these poor survival statistics, the possible benefit of adjuvant chemotherapy has been investigated in several clinical trials. The BILCAP trial—a randomized, controlled, multicenter phase III study—performed across 44 specialized centers in the UK compared OS between patients who received adjuvant capecitabine vs. observation. Patients with BTC who underwent surgical resection with curative intent were randomly assigned to the capecitabine group (*n* = 223) or the observation group (*n* = 224). The median follow-up for patients was 60 months. In the intention-to-treat analysis, median OS was 51.1 months in the capecitabine group compared with 36.4 months in the observation group. However, in a protocol-specified sensitivity analysis that adjusted for minimization factors and nodal status, grade, and gender, the OS hazard ratio for the capecitabine group was 0.71 (95% CI 0.55–0.92; *p* = 0.010) [72]. Additionally, in the per-protocol analysis, median OS was 53 months in the capecitabine group compared to 36 months in the observation group. Capecitabine treatment was tolerated by most patients, and there were no treatment-related deaths. This study provided evidence that adjuvant capecitabine is tolerated and can improve OS in patients following surgical resection.

The effectiveness of GC vs. capecitabine as adjuvant chemotherapy after surgical resection of extrahepatic CCA was also examined. These patients had undergone curative-intent surgical resection but were found to have regional lymph node metastases. They were randomized to receive GC (*n* = 50) or capecitabine (*n* = 51), with a primary endpoint of DFS. There was no statistically significant difference in 2-year DFS rates between the GC and capecitabine groups. Median OS was 35.7 months for both groups. Grade 3–4 adverse events occurred in 84% of patients who received GC, compared to 16% in those who received capecitabine. There were no treatment-related deaths in either group [80]. These results illustrate that adjuvant GC did not improve survival outcomes when compared with capecitabine, with more significant adverse events. Adjuvant chemotherapy after surgical resection has demonstrated a potential survival benefit and further investigations should continue.

### 2.4. Addition of Neoadjuvant Chemotherapy

Most patients with resectable CCA undergo surgical resection followed by adjuvant chemotherapy. However, there is limited survival benefit seen with this current practice pattern, especially for patients with high-risk characteristics. Administering chemotherapy in the neoadjuvant setting offers a potential opportunity to improve the outcomes of patients with resectable disease. The addition of nab-paclitaxel to gemcitabine and cisplatin (GAP) has demonstrated a high disease control rate and improved survival outcomes. Hence, this regimen was given to patients prior to curative-intent surgery for resectable, but high-risk, iCCA. This NEO-GAP study was a single-arm, phase II trial with 30 patients enrolled. Twenty-two patients completed all preoperative chemotherapy and underwent surgical resection. There was no treatment-related mortality, and only 10 patients experienced grade 3 or above treatment-related adverse events, the most common being neutropenia and diarrhea. Two of the patients who underwent surgical resection had minor postoperative complications, with a median length of hospital stay of 4 days. The disease control rate was 90% and median RFS was 7.1 months [73]. This study illustrated that neoadjuvant treatment with GAP is reasonable and safe prior to the resection of iCCA, and it does not adversely affect perioperative outcomes. Efficacy was not the primary endpoint of this trial, so further studies are needed to examine the potential survival benefits of neoadjuvant chemotherapy in resectable CCA.

### 2.5. Chemotherapy Limitations—Development of Chemoresistance

Although retrospective reviews and clinical trials demonstrate encouraging results regarding increased survival with several chemotherapy regimens, the median OS for patients with CCA is only between 11 and 15 months [81]. The modest efficacy and short duration of tumor response to chemotherapy is likely due to the development of chemoresistance, especially multidrug resistance. Multidrug resistance results from defense mechanisms against toxic compounds that are already present in cholangiocytes. These natural defense mechanisms plus mechanisms of chemoresistance (MOCs) are often present with enhanced efficacy in tumors derived from cholangiocytes [82]. MOCs often act synergistically to allow cancer cells to escape the toxic effects of cytostatic drugs, including most chemotherapy drugs.

For example, the “resistome” has been defined as the entire set of MOC-associated proteins that are expressed in tumor cells. In CCA specifically, seven categories of MOC have been identified, including: enhanced drug export (MOC-1b), enhanced DNA repair (MOC-4), reduced apoptosis (MOC-5a), and enhanced survival (MOC-5b). Upregulation of MOC-4 genes involved in DNA repair after chemotherapy exposure is a common feature in CCA. For example, the excision repair cross-complementing protein 1 (ERCC1) was found to be upregulated in CCA cells after treatment with cisplatin. The overexpression of ERCC1 has been suggested as a potential prognostic marker in patients with advanced CCA after cisplatin treatment [83]. Additionally, the expression level of human equilibrative nucleoside transporter 1 (hENT1), the major gemcitabine transporter identified in cancer cells, has been correlated with their sensitivity to gemcitabine. CCA cells that developed gemcitabine resistance have downregulation of hENT1 and upregulation of multidrug resistance protein 1 (MRP1), causing increased efflux of gemcitabine from cells. These cells also evaded apoptosis via upregulation of B-cell lymphoma 2 (BCL2) and downregulation of BCL2-associated X, apoptosis regulator (BAX) [81].

ATP-binding cassette (ABC) transporters are a large family of membrane proteins that play an important role in the efflux of chemotherapeutic agents from cells [84]. Overexpression of ABC transporters is associated with tumor aggressiveness, progression, and poor patient prognosis in several cancer types [85]. The overexpression of ABC transporters plays a significant role in multidrug resistance (MDR) and is associated with poor prognosis in CCA patients. In CCA patients, multidrug resistance-associated protein 1 (MRP1 or ABCC1) is overexpressed compared to normal tissues. The overexpression of MRP1 was also found to be significantly correlated with shortened OS in CCA patients [86]. This is one of the several mechanisms utilized by cancer cells to promote chemotherapy resistance. Further understanding and identifying these mechanisms in CCA cells is important for developing novel strategies to overcome chemoresistance, including targeted therapies.

## 3. Targeted Therapies

Chemotherapy remains the standard treatment modality for unresectable CCA. However, due to the aggressive nature of CCA and the development of chemoresistance, more targeted therapies are used in conjunction with or in place of chemotherapy. Tissue biopsies and cytology samples are utilized with FISH and NSG to detect common mutations and identify potential gene targets for more personalized therapy. Although iCCA and eCCA subtypes are often managed using similar treatment approaches, they differ in their underlying tumor etiology and molecular profiles [87]. For example, integrated genomic, epigenetic, and transcriptomic analyses were performed on CCA subtypes, and four clusters were recognized with their genetic, epigenetic, and clinical features [11]. Cluster 1 is associated with human epidermal growth factor receptor tyrosine-protein kinase erbB-2 (*HER2*) amplification and CpG island hypermethylation; cluster 2 is associated with *TP53* mutations and *HER2* amplification; cluster 3 is associated with upregulated immune-related pathways (e.g., programmed cell death protein 1 (PD-1)); and cluster 4 is associated with *BAP1* mutations, *IDH1/2* mutations, and fibroblast growth factor receptor (*FGFR*) alterations while being found mostly in iCCA. These clusters are also driven by different etiologies; clusters 1 and 2 are frequently associated with liver fluke infections, while clusters 3 and 4 are not associated with these infections [87]. These findings and other discoveries have resulted in the identification of potential therapeutic targets for CCA, including FGFR2, HER2, IDH1, and PD-1 (Table 2).

### 3.1. FGFR Inhibitors

Activating alterations of *FGFR2* are found in 12 to 15% of iCCA cases, with the most common alteration causing fusion of the *FGFR2* gene to various partners [89]. More than 150 fusion partners have been identified, with variable frequency. Appropriately regulated FGFR signaling plays a significant role in various cellular processes, including proliferation, survival, and angiogenesis, while dysregulated FGFR activity can cause malignant transformation of cells [90]. *FGFR2* fusions have been identified as key factors that drive aerobic glycolysis in iCCA cases. Inhibition of FGFR impairs glucose metabolism, which limits the adaptability of iCCA cells and creates targetable metabolic vulnerabilities for potential pharmacologics [89]. Genomic data show that approximately 40% of patients with CCA have potentially targetable genetic mutations. These findings underscore the importance of comprehensive genomic testing to identify possible personalized treatments for patients with CCA [91]. Retrospective analyses demonstrated that FGFR inhibitors were associated with improved survival compared with standard second-line chemotherapies [92].

Selective FGFR inhibitors have been developed as targeted therapy for patients with *FGFR* alterations. Pemigatinib is a selective FGFR1-3 inhibitor that was the first targeted therapy to receive U.S. Food and Drug Administration (FDA) approval for the treatment of CCA. The Phase II FIGHT-202 study assessed the efficacy and safety of pemigatinib in patients with locally advanced or metastatic CCA who had progressed after at least one prior systemic therapy. In 107 patients with *FGFR2* fusions or rearrangements, pemigatinib yielded an objective response rate (ORR) of 35.5% with three complete responses, a median PFS of 6.9 months, and median OS of 21.1 months. There were no responses to pemigatinib in patients without *FGFR2* fusions or rearrangements [91]. Currently, there are several FGFR-selective inhibitors being tested in clinical trials for patients with CCA, including pemigatinib, derazantinib, futibatinib, and infigratinib (Table 3). These studies have demonstrated clinical benefit of FGFR inhibitors as second-line treatment for patients with advanced CCA.

### 3.2. IDH1 Inhibitors

IDH1 is an enzyme that catalyzes the oxidative decarboxylation of isocitrate to α-ketoglutarate (αKG) while reducing NADP^+^ to NADPH. Mutated *IDH1* results in the abnormal accumulation of the oncometabolite 2-hydroxyglutarate (2-HG), which triggers tumorigenesis by competitively inhibiting other enzymes that regulate DNA repair, cellular metabolism, and epigenetics [106]. The reported frequency of *IDH1* mutations in iCCA is 13.1% vs. 0.8% in eCCA [107]. Systematic reviews indicate that the prevalence of mutated *IDH1* in patients with iCCA in the U.S. is 18.0%. However, none of the studies reported a statistically significant association between mutated *IDH1* and OS, PFS, or time to disease progression [108].

Ivosidenib is a targeted inhibitor of mutated *IDH1* that was given to patients with histologically confirmed, advanced, *IDH1*-mutant CCA in a multicenter, randomized, phase III clinical trial. The primary endpoint was PFS, with secondary endpoints, including OS, ORR, and duration of response. One hundred and eighty-five patients were randomly assigned to the ivosidenib group (*n* = 124) or placebo group (*n* = 61). PFS was significantly longer (*p* < 0.0001) for patients in the ivosidenib group (2.7 months) compared to the placebo group (1.4 months). PFS was 32% at 6 months and 22% at 12 months in the ivosidenib group, whereas no patients in the placebo group remained progression-free for 6 months or longer. Ivosidenib was well-tolerated with minimal significant adverse events and no treatment-related deaths. This study showed the clinical benefit of targeted therapies against genetic mutations commonly found in cases of CCA [97].

### 3.3. HER2 Inhibitors

HER2 overexpression drives uncontrolled cellular signaling through activation of downstream cascades, including proliferation pathways and pro-survival pathways [109]. HER2 amplification has been utilized as a predictive biomarker for HER2-targeted agents in various cancer types, including breast and gastric cancer [110]. HER2 overexpression is more frequent in eCCA, occurring in 19.9% of cases, compared to 4.8% in iCCA [109]. Retrospective dataset analyses have shown that *HER2* amplifications or mutations are associated with poor OS in patients with metastatic BTC, while HER2 overexpression was associated with an increased risk of disease recurrence in patients with surgically resected BTC [100].

HER2 inhibitors such as pertuzumab and trastuzumab have demonstrated improved patient outcomes in other cancer types, including breast, gastric, and esophageal cancer. The MyPathway study, a multicenter phase II trial, included patients with previously treated HER2-positive metastatic biliary cancer who were treated with pertuzumab plus trastuzumab as dual anti-HER2 therapy. Nine of the 39 patients had an objective response, yielding an ORR of 23%, while nine patients had stable disease for up to 4 months. The PFS was 4.0 months, with OS of 10.9 months. There were no treatment-related serious adverse events or treatment-related deaths. These data show that HER2 inhibitors are well-tolerated in patients and have potential as targeted therapies for patients with HER2-positive CCA [98]. Currently, there are several HER2 inhibitors utilized in trials, including pertuzumab, trastuzumab, tucatinib, and neratinib (Table 4).

### 3.4. PD-1/PD-L1 Inhibitors

The inhibitory co-receptor PD-1 and its ligand PD-L1 have been recognized as key mediators of immunosuppression within a tumor microenvironment (TME). When PD-1 binds to PD-L1, it inhibits immune responses, thereby allowing tumor cells to escape immune-mediated destruction. Upregulation of PD-L1 seen in various tumor types has been associated with increased tumor invasion and poor patient outcomes. One study examined PD-L1 expression in iCCA cases by performing immunohistochemistry (IHC) on fixed tumor samples from patients who underwent surgical resection. Thirty-nine of the 54 tumor samples (72%) stained positive for PD-L1 expression on cells within the tumor front. Tumor characteristics like nodal metastasis and number of lesions were significantly associated with the upregulation of PD-L1. Patients with PD-L1 expression within the tumor front showed a 60% decrease in survival. These findings highlight the critical role of PD-L1 in tumor progression, making it a valuable therapeutic target for CCA [111].

Several PD-1/PD-L1 inhibitors have shown durable antitumoral effects in various solid tumors, including melanoma, renal cell carcinoma, and non-small cell carcinoma [111]. Pembrolizumab, a monoclonal antibody that targets and blocks PD-1, has been approved for the treatment of advanced lung cancer after progression on first-line therapy [112]. The KEYNOTE-966 study was a randomized, double-blind phase III trial for patients with locally advanced or metastatic biliary cancer who received either pembrolizumab plus GC (*n* = 533) or placebo plus GC (*n* = 536). The primary endpoint was OS in the intention-to-treat population. Median OS was 12.7 months in the pembrolizumab group compared to 10.9 months in the placebo group (hazard ratio 0.83, *p* = 0.0034). Median PFS was 6.5 months in the pembrolizumab group and 5.6 months in the placebo group. Both groups had treatment-related adverse events, including eight (2%) treatment-related deaths in the pembrolizumab group and three (1%) in the placebo group [68]. Treatment-related adverse reactions, including cholestasis and cholangitis, have also been observed with nivolumab [113]. Therefore, patients who receive these molecules require close observation and monitoring. There are several PD-1/PD-L1 inhibitors currently in clinical trials for treatment of CCA, including pembrolizumab [103], durvalumab [102], and nivolumab [104]. These antibodies have demonstrated clinically significant improvements in OS, especially when combined with chemotherapy [68,70,105].

### 3.5. Targeted Therapy Limitations

As novel systemic therapies have entered clinical practice, their limitations have been recognized. These include the technical challenges and resources needed for identifying biomarkers to select targeted therapies for patients. Additionally, these therapies need to overcome primary and secondary drug resistance, often in combination with other systemic drugs. The heterogeneity in the expression levels of molecular targets may predict the lack of efficacy of certain drugs [114]. Additionally, in response to drug exposure, cancer cells may alter the expression levels of genes and proteins involved in resistance mechanisms [115,116]. The mutations that affect the activity levels of these proteins can also induce cross-resistance to different drugs [117]. New mutations in tyrosine kinase receptors and other targets are a method of acquired resistance to targeted agents [115]. These mechanisms can be responsible for disease progression after an initial response. For example, decreased expression of PD-LI has been associated with worse outcomes in patients with CCA who were previously treated with monoclonal antibodies targeting PD-1, such as nivolumab or pembrolizumab [104,118].

Resistance to FGFR inhibitors has been examined based on their mechanism of action. The activity of FGFR receptors depends on the binding to the ATP-binding pocket region on the tyrosine kinase domain. This region is called the “gatekeeper” because any structural change can reduce the specificity and activity of the FGFR inhibitors. The main gatekeeper of the FGFR protein is a valine residue, which is prone to secondary mutations after being exposed to continuous FGFR inhibition [119,120]. Additionally, continuous FGFR inhibition can lead to the upregulation of alternative receptors in tumor cells, resulting in reduced dependence on the FGFR-signaling pathway [121]. Strategies to overcome resistance include combination therapy with other antineoplastic drugs with different mechanisms of action and the development of more specific and potent drugs targeting individual pathways [122].

## 4. Emerging/Investigational Therapies

### 4.1. Antibody-Drug Conjugates

Antibody-drug conjugates (ADCs) are a novel therapy designed to selectively attack cancer cells while sparing normal cells. These conjugates are composed of three elements: (1) Antibody—provides specificity by selectively binding to the target cell receptor; (2) Linker—connects the antibody to the payload; and (3) Payload—cytotoxic compound that is internalized by the target cell, leading to cell death [123]. Trastuzumab deruxtecan (T-DXd) is one such ADC consisting of a monoclonal anti-HER2 antibody, a cleavable tetrapeptide-based linker, and a potent topoisomerase I inhibitor as the payload. The linker is stable in the plasma, but following internalization, it is selectively cleaved by lysosomal enzymes such as cathepsins, which are overexpressed in the TME [124]. The payload is released once the linker is cleaved.

T-DXd has been administered to patients in various clinical trials for several different cancer types, including breast cancer, gastric cancer, and colorectal cancer [124]. The DESTINY-PanTumor02 trial, a multicenter phase II study, examined the efficacy and safety of T-DXd in patients with treatment-refractory HER2-overexpressing ([IHC] 2^+^/3^+^) solid tumors. Patients were divided into seven cohorts, based on cancer type. One of the cohorts consisting of 41 patients with HER2-expressing BTC showed an ORR of 22.0% to T-DXd treatment. This group was further separated based on levels of HER2 expression seen in IHC. Patients with 3^+^ HER2 expression had an ORR of 56.3% and median OS of 12.4 months [125]. This study showed the clinical benefits of T-DXd treatment in patients with HER2-overexpressing BTC.

Several clinical trials for ADCs are currently underway or actively recruiting patients with BTC. MRG003 is an ADC composed of an anti-epidermal growth factor receptor (EGFR) monoclonal antibody that is connected to monomethyl auristatin E. It is being used in a multicenter phase II trial for the treatment of *EGFR*-positive unresectable or metastatic BTC refractory to standard therapies (NCT04838964). Additionally, AZD8205 contains an anti-B7-H4 antibody coupled to a topoisomerase I inhibitor payload. It is being used in a multicenter phase I/II trial as a monotherapy or in combination with other treatment options for patients with advanced or metastatic solid tumors, including BTCs (NCT051203482) [123]. These studies will provide further information regarding the safety and efficacy of ADCs in patients with treatment-resistant or advanced BTC.

### 4.2. CAR T Cells

Downregulation of major histocompatibility complex (MHC) molecules is a key mechanism by which tumor cells evade immune responses. The development of chimeric antigen receptor (CAR) T cells provides a method to prevent tumor escape from the immune system [126]. CAR T cells are genetically engineered T cells that recognize tumor-associated antigens (TAAs) expressed on cancer cells [126] and induce apoptosis based on an MHC-independent recognition mechanism [127]. CAR T cells are comprised of an extracellular single-chain variable fragment (scFV) for antigen recognition, a transmembrane domain, and intracellular signaling domains [126]. Different generations of CAR T cells have been developed by combining the intracellular component of the T cell receptor (CD3ζ) and one or more co-stimulatory domains [127]. Multiple generations of CAR T cells have been formulated to insert several co-stimulatory domains into the CAR molecule to improve efficacy and persistence. Second-generation CD19 CAR T cells have been approved by the FDA for the treatment of hematologic malignancies [127]. Similar CAR T cell therapies for patients with solid tumors have been less successful, prompting continued development and modification to these molecules [128].

The novel fourth-generation CAR (CAR4) T cells contain three costimulatory molecules CD28/CD137/CD27 and have been shown to be both safe and effective for patients with acute lymphoblastic leukemia, lymphoma, and sarcoma. Therefore, these CAR4 T cells have the potential to be utilized for the treatment of CCA. Although a unique antigen has not been identified in CCA, CD133 has emerged as a potential surface biomarker. CD133 is also defined as a cancer stem cell (CSC) marker associated with tumor metastasis, making it a potential antigen to target with CAR T cells [126]. IHC has revealed high expression of CD133 in 67.6% of CCA specimens. CD133 expression in patients with CCA is also associated with poor prognosis and higher recurrence rate compared to the CD133-negative population. Anti-CD133 CAR4 T cells effectively killed CD133-expressing CCA cells in culture and in a three-dimensional spheroid model [126]. This study demonstrated the potential of anti-CD133 CAR4 T cells to target CD133-positive CCA in the preclinical setting. Further studies are needed to test the safety and efficacy of anti-CD133 CAR4 T cells in the clinical setting.

EGFR overexpression has been noted in both iCCA and eCCA, making it a potential marker for targeted therapies. However, EGFR inhibitors such as erlotinib have not significantly improved OS or PFS in patients with BTC. EGFR-specific CAR T cells have been engineered and were well-tolerated and effective in patients with EGFR-positive advanced non-small cell lung cancer. This study also demonstrated that conditioning chemotherapy given before CAR T cell infusion leads to better clinical outcomes compared to CAR T cell therapy alone [129]. A phase I study was initiated for patients with unresectable or relapsed EGFR-positive BTC in which 19 patients received nab-paclitaxel and cyclophosphamide as conditioning chemotherapy followed by 1–3 cycles of anti-EGFR CAR T cells. The CAR T cell infusions were tolerated, with only three patients experiencing grade ≥ 3 fever/chills. Grade 1/2 infusion-related toxicities, including mucosal/cutaneous reactions and acute pulmonary edema, were observed in eight patients. One patient achieved a complete response, and 10 patients experienced stable disease after treatment. The median PFS was 4 months from the first cycle of treatment [130]. This study illustrated that anti-EGFR CAR T cell therapy is safe for patients, with manageable side effects and the potential for clinically significant outcomes when combined with chemotherapy for patients with advanced BTC.

### 4.3. Bispecific T Cell Engagers in the Preclinical Setting

Bispecific T cell engagers are engineered proteins made of two single-chain variable fragments that are fused to form bi-functional domains; one domain targets TAAs on tumors cells, and the other domain targets CD3 chains on T cells [131]. Bispecific T cell engagers can guide T cells to engage with tumor cells expressing unique TAAs, which initiates tumor cell lysis. In 2017, the FDA approved the first bispecific T cell engager (αCD19/αCD3) for the treatment of relapsed or refractory B-acute lymphoblastic leukemia (B-ALL). Currently, there are several bispecific T cell engagers involved in clinical trials for hematologic malignancies and solid tumors [131]. In the pre-clinical setting, a PD-L1xCD3 bispecific T cell engager was created to target PD-L1 on tumor cells while binding to CD3 on T cells to cause T cell activation and cancer cell killing [132]. Prior studies demonstrated that gemcitabine treatment stimulated PD-L1 expression in CCA cell lines and boosted T cell cytotoxicity [133]. In this study, PD-L1xCD3 bispecific T cell engager treatment significantly enhanced T cell cytotoxicity against CCA cells in vitro, particularly after gemcitabine treatment. Additionally, higher PD-L1 expression increased the magnitude of cancer cell killing [132]. These results indicate that the combination treatment of gemcitabine and PD-L1xCD3 bispecific T cell engagers may be a potential therapy for CCA.

Despite these results, the short half-life of bispecific T cell engagers necessitates continued infusions, which can lead to severe side effects and substantial costs [134]. A strategy to overcome these limitations is to arm T cells with bispecific T cell engagers. These engineered cells require a significantly smaller amount of bispecific T cell engager compared to bispecific T cell engager treatment alone, potentially diminishing the severity of side effects. Clinical trials have shown the efficacy of bispecific T cell engager-armed T cells with the noteworthy absence of dose-limiting toxicities for treating both hematologic and solid malignancies [134]. In the pre-clinical setting, genetically modified T cells that secrete αCD133-αCD3 bispecific T cell engagers were created by transducing T cells separated from peripheral blood mononuclear cells (PBMCs) with lentiviruses carrying cDNA encoding the αCD133-αCD3 bispecific T cell engagers. The killing ability of these modified T cells was tested in CD133-expressing CCA cells. The modified T cells demonstrated significantly higher cytotoxicity against CCA cells in vitro than non-modified T cells. Additionally, the secreted αCD133-αCD3 bispecific T cell engager stimulated both the transduced T cells and bystander T cells to kill the CCA cells [131]. These modified T cells that secrete αCD133-αCD3 bispecific T cell engagers may be a novel treatment for CD133-positive CCA.

Another group engineered T cells to secrete an A20/αCD3 bispecific T cell engager, which targets integrin αvβ6 on cancer cells and CD3 on T cells. Integrin αvβ6 is an antigen that is elevated in various malignancies, including CCA. In pre-clinical studies, the A20/αCD3-armed T cells showed significantly increased killing against integrin αvβ6-expressing CCA cells and spheroids compared to unarmed T cells. These A20/αCD3-armed T cells exhibited elevated T cell activation markers, improved T cell proliferation, and increased cytokine production when compared to unarmed T cells [134]. Bispecific T cell engager-armed T cells offer a potential therapeutic benefit by combating immune evasion and co-activating T cells, thereby generating a more robust immune response against cancer cells. Further studies are required to examine their safety and efficacy in the clinical setting.

### 4.4. Oncolytic Viruses

The use of genetically modified viruses as oncolytic agents has emerged as a novel therapy against cancer. Several viruses, such as adenovirus, West Nile virus (WNV), herpes simplex virus (HSV), and vaccinia virus (VACV), have demonstrated potential as OVs as they successfully infect, replicate in, and kill cancer cells [135]. Furthermore, OVs have exhibited the ability to reprogram the “cold” TME to “hot” and activate antitumor immune responses by reprogramming macrophages and activating innate immune cells [136,137]. OVs have advantages over conventional therapies due to their tumor selectivity and unique killing ability [138]. Selectivity through genetic modification is often achieved by disrupting viral genes that are essential for replication in normal cells but dispensable in cancer cells. A common example is the deletion of the *J2R* gene, which encodes thymidine kinase (TK), in vaccinia virus. Since cancer cells typically express high levels of TK, the loss of *J2R* has minimal impact on viral replication in these cells. In contrast, non-dividing normal cells have very low TK levels, resulting in attenuation of the *J2R*-deleted vaccinia virus in normal tissues [138]. GLV-1h68 is a replication-competent oncolytic VACV with disruptions in the *J2R*, *F14.5L*, and the *hemagglutinin* genes that has shown oncolytic activity in three human CCA cell lines [135]. Additionally, a single dose of GLV-1h68 significantly suppressed CCA tumor growth in subcutaneous flank xenografts in athymic nude mice. Phase I clinical trials of GLV-1h68 have been completed and further studies may demonstrate clinical benefits for CCA [135].

Another group generated a unique strain of VACV called CVV, which was engineered with deletion of TK and insertion of green fluorescence protein (GFP) and guanine-hypoxanthine phosphoribosyltransferase (GPT) selection markers [138]. In the pre-clinical setting, the CVV virus successfully infected and killed CCA cells in vitro. Additionally, athymic nude mice with CCA xenografts were injected intraperitoneally with the CVV virus, and tumors and organs were collected 2 weeks later. Significant CVV levels were observed exclusively in the tumors with negligible levels in the organs, demonstrating the selective oncolytic activity of the virus against CCA. Furthermore, the CVV virus combined with gemcitabine or cisplatin demonstrated increased cytotoxicity against CCA cells in vitro when compared to either drug alone [138]. OVs, such as VACV, may enhance therapeutic benefits when combined with standard chemotherapy and can potentially overcome drug resistance commonly observed in CCA.

Vaccinia OVs have been used in clinical trials, including CF33-hNIS, which is a novel chimeric poxvirus encoding the human sodium-iodide symporter (*hNIS*) gene [139]. The MAST study evaluated the safety of CF33-hNIS administered intratumorally (IT) or intravenously (IV), alone or combined with pembrolizumab in patients with advanced or metastatic gastrointestinal tumors who failed ≥ 2 prior lines of therapy. Two of the seven patients who received CF33-hNIS alone had bile duct cancer. One of these two patients achieved a complete response to IT CF33-hNIS by the fourth cycle with no observed cancer recurrence. The other patient received IV-administered OV and achieved stable disease for >5 months. There were no dose-limiting toxicities, and there were minimal treatment-related adverse events following OV monotherapy [139]. Combination of CF33-hNIS and pembrolizumab is currently being evaluated for safety and efficacy in advantaged stage gastrointestinal tumors, including CCA. Findings from these trials will indicate the efficacy of OVs in combination with other drugs in the clinical setting.

## 5. Clinical Trial Limitations

There are currently numerous clinical trials evaluating novel treatment regimen for CCA patients, with a recent focus on targeted therapies and immunotherapy in combination with other modalities. Many of these clinical trials are phase I and II clinical trials, with small sample sizes. Therefore, further research with larger patient cohorts, multicenter projects, and higher quality is necessary [140]. The safety and efficacy of these various combination therapies need to be evaluated in these larger clinical studies. This includes identifying any treatment-related adverse events and recognizing how to manage them. Furthermore, it is essential to consider the prices of these drugs for patients. There could be differences in efficacy due to treatment selection based on affordability [140]. Additionally, improving CCA treatment through precise molecular profiling to identify sensitivity to targeted therapies is important. The combination of traditional and personalized novel treatments can improve the effectiveness of CCA treatment.

A major challenge encountered in CCA research is the absence of uniformity in categorizing its subtypes [141]. Additionally, CCA patients and gallbladder cancer patients are frequently combined into one cohort. Clinical trials may also include patients with liver cancers and BTCs in the same group. It is difficult to study CCA patients when outcomes are being generalized for larger, non-uniform cohorts in these trials [142]. Additionally, representation of all subpopulations in clinical trials is critical as the incidence and risk factors for CCA vary between countries. However, Hispanic and non-Hispanic black populations were under-represented in global phase III clinical trials for liver cancer and CCA from 2012 to 2022. Of the 17 studies analyzed, only eight reported ethnicity data, which may contribute to disparities in treatment [143]. Without representation of the whole population in clinical trials, treatment guidelines will not be accurate or effective.

## 6. Conclusions

CCA is a rare malignancy, but its aggressive nature and often late-stage presentation make it a challenging disease to treat. The only curative treatment is surgical resection, but most patients are not surgical candidates due to the advanced stage of disease at diagnosis. Chemotherapy is a mainstay treatment strategy, with several recent advances, including identification of optimal first-line chemotherapy regimens and other emerging second-line or third-line therapies. Neoadjuvant chemotherapy has the potential to convert borderline resectable tumors to resectable tumors, allowing more patients to become eligible for surgery. However, chemotherapy resistance is not uncommon and numerous patients fail 1st-, 2nd-, and 3rd-line therapies. More recent advances in genetic testing have identified specific mutations within tumors, which have been targeted for more personalized therapies. Antibodies and ADCs have been formulated to inhibit these aberrant driver proteins, including FGFR2, HER2, IDH1, and PD-L1. Additionally, CAR T cells and bispecific T cell engagers have been engineered to target specific TAAs to overcome immune evasion mechanisms by cancer cells. OVs have been designed to exclusively replicate in and lyse cancer cells while leaving normal cells unaffected. Personalized and targeted therapies show strong clinical promise against CCA, with several currently under investigation in clinical trials. Continued research is essential to refine existing treatments and inform clinical trials to accelerate development of effective therapies for CCA.

## Figures and Tables

**Table 1:** Completed chemotherapy trials for solid tumors including cholangiocarcinoma patients.

Trial	Therapy	Condition	Outcome	Adverse Events (AEs)
Phase III (ABC-02) NCT00262769	Gemcitabine (G) + Cisplatin (C) [67]	Locally advanced or metastatic CCA, GBC, or ampullary cancer	Median OS 11.7 months in G + C vs. 8.1 months for G alone Median PFS 8.0 months in G + C vs. 5 months in G alone	No difference in grade 3/4 toxic effects for G + C group vs. G alone No difference in grade 3/4 neutropenia for G + C group vs. G alone
Phase III (KEYNOTE-966) NCT04003636	Gemcitabine (G) + Cisplatin (C) + Pembrolizumab (P) [68]	Untreated, unresectable, locally advanced or metastatic BTC	Median OS 12.7 in G + C + P vs. 10.9 months in placebo	Similar rates of grade 3/4 AEs for G + C + P vs. placebo Treatment-related AEs led to death in 2% of G + C + P patients vs. 1% in placebo
Phase III (ABC-06) NCT01926236	FOLFOX (after Gemcitabine + Cisplatin progression) [69]	Locally advanced or metastatic BTC (including CCA and gallbladder or ampullary carcinoma)	Median OS 6.2 months in FOLFOX vs. 5.3 in control	Grade 3–5 AEs in 69% of FOLFOX group vs. 52% in control Three chemotherapy-related deaths in FOLFOX group
Phase III (TOPAZ-1) NCT03875235	Gemcitabine (G) + Cisplatin (C) + Durvalumab (D) [70]	Untreated, unresectable, or metastatic BTC	Median OS 12.8 months in D + G + C vs. 11.5 months in G + C Median PFS 7.2 months D + G + C vs. 5.7 months in G + C	Similar rates of grade 3/4 AEs for D + G + C vs. G + C Grade 3/4 immune-mediated adverse effects were 2.4% for D + G + C vs. 1.5% in G + C
Phase III (SWOG S1815) NCT03768414	Gemcitabine (G) + Cisplatin (C) vs. G + C + Nab-paclitaxel (GAP) [71]	Newly diagnosed locally advanced unresectable or metastatic BTC, including iCCA and eCCA, and GBC	No OS benefit Median OS with GAP 14.0 months vs. 13.6 months in G + C Median PFS with GAP 7.5 vs. 6.3 with G + C	Grade 3/4 AEs in 41% of GAP group vs. 28% in G + C Grade 3/4 hematologic AEs 60% in GAP vs. 45% in G + C
Phase III (BILCAP) 2005-003318-13	Capecitabine (Adjuvant) [72]	Histologically confirmed CCA or muscle-invasive GBC	In intention-to-treat analysis, median OS 51.1 months in capecitabine group vs. 36.4 months in observation group In per-protocol analysis, median OS 53 months in capecitabine group vs. 36 months in observation group	52% of the serious adverse effects in capecitabine group were related to treatment 8% of AEs were cardiac events related to capecitabine
Phase III (NEO-GAP)	Neoadjuvant GAP (gemcitabine, cisplatin, and nab-paclitaxel) [73]	Resectable, high-risk iCCA	DCR was 90% 73% completed all chemotherapy and surgery Median OS 24 months	33% of GAP patients experienced ≥grade 3 AEs Neutropenia was most common ≥grade 3 AE (17% of patients)
Phase II (GEMOX)	Gemcitabine-oxaliplatin (GEMOX) (Adjuvant) [65]	ABTA	Group A patients had no history of prior chemotherapy or radiotherapy PFS 5.7 months OS 15.4 months Group B patients had prior chemotherapy or radiotherapy treatments Median PFS 3.9 months Median OS 7.6	Neutropenia most common grade 3/4 toxicity in GEMOX group (14% of patients) No toxicity-related deaths
Phase Ib/II (BilT03) NCT03785873	Nivolumab + Nanoliposomal-Irinotecan, 5-Fluorouracil, and Leucovorin [74]	Second line therapy for patients with advanced BTC	Median PFS 4.1 months Median OS 7.4 months	Most common ≥grade 3 AEs were diarrhea (16.7%), fatigue (13.3%), and neutropenia (10%) Two treatment-related deaths
Phase II/III (TreeTopp) NCT03093870	Varlitinib (V) + Capecitabine (C) vs. Placebo + Capecitabine (C) [75]	BTC	Median PFS 2.83 V + C versus 2.79 months for placebo + C OS 7.8 versus 7.5 months	65.6% of V + C patients with ≥grade 3 AEs vs. 58.7% in placebo + C Three patients in V + C with AEs leading to discontinuation

BTC: Biliary tract cancer; CCA: cholangiocarcinoma; iCCA: intrahepatic cholangiocarcinoma; eCCA: extrahepatic cholangiocarcinoma; GBC: gallbladder cancer; ABTA: Advanced biliary tract adenocarcinoma; OS: overall survival; PFS: progression free survival; DCR: disease control rate; AE: adverse events.

**Table 2:** Cholangiocarcinoma subtype-associated gene mutations.

iCCA Subtype [88]		eCCA Subtype [88]	
Gene Mutations	Percentage	Gene Mutations	Percentage
*TP53*	27–40%	*TP53*	40%
*IDH1/2*	13–20%	*IDH1/2*	<1%
*FGFR1/2/3*	12–15%	*FGFR1/2/3*	<1%
*KRAS*	12%	*KRAS*	28–36%
*BRAF*	3–10%	*BRAF*	3%
*HER2 (ERBB2)*	2–7%	*HER2 (ERBB2)*	15–20%

*TP53*: tumor protein 53; *IDH1/2*: isocitrate dehydrogenase 1/2; *FGFR1/2/3*: fibroblast growth factor receptor 1/2/3; *KRAS*: Kirsten rat sarcoma virus; *BRAF*: B-raf proto-oncogene, serine/threonine kinase; *HER2*: human epidermal growth factor receptor 2; *ERBB2*: erythroblastic oncogene B 2.

**Table 3:** Summary of the targeted therapies for cholangiocarcinoma (CCA) treatment.

Targeted Therapies for Treatment of CCA			
**Medication**	Mechanism	Trial (s)	Results
FGFR-targeted therapy			
Pemigatinib	Selective FGFR1-3 inhibitor [91]	Phase II (FIGHT-202)	ORR 35.5% Median PFS 6.9 months
Debio 1347	Selective FGFR1-3 inhibitor [93]	Phase II (FUZE)	Median PFS 3.68 months PR in 2/30 patients (7%)
Derazantinib	Pan-FGFR inhibitor [94]	Phase I/II (NCT01752920)	ORR 20.7% DCR 82.8% Median PFS 5.7 months
Futibatinib	Selective FGFR1-4 inhibitor [95]	Phase II (FOENIX-CCA2)	ORR 42% Median PFS 9.0 months Median OS 21.7 months
Infigratinib	Selective FGFR1-3 inhibitor [96]	Phase II (NCT02150967)	ORR 23.1% DCR 84.3% Median PFS 7.3 months
IDH1-targeted therapy			
Ivosidenib	Targeted inhibitor of mutated IDH1 [97]	Phase III (ClarIDHy)	Median PFS 2.7 months PFS significant improvement when compared with placebo (*p* < 0.0001)
HER2-targeted therapy			
Pertuzumab plus Trastuzumab	HER2 dimerization inhibitor; HER2 inhibitor [98]	Phase II (MyPathway)	ORR 23.0% Median PFS 4.0 months Median OS 10.9 months
Tucatinib plus Trastuzumab	HER2-specific tyrosine kinase inhibitor; HER2 inhibitor [99]	Phase II (KCSG-HB19-14)	ORR 46.7% DCR 76.0% PFS 5.5 months
Neratinib	Pan-HER tyrosine kinase inhibitor [100]	Phase II (SUMMIT)	ORR 16.0% DCR 24.0% Median PFS 2.8 months
Trastuzumab plus FOLFOX	HER2-specific tyrosine kinase inhibitor (Trastuzumab) [101]	Phase II (SGNTUC-019)	ORR 29.5% DCR 79.4% Median PFS 5.1 months
PD-1/PD-L1 Inhibitors			
Durvalumab	PD-L1 inhibitor [102]	Phase I (NCT01938612)	ORR 4.8% Median PFS 1.4 months Median OS 8.1 months
Pembrolizumab	PD-1 inhibitor [103]	Phase II (NCT03110328)	ORR 10%, DCR 37.5% Median PFS 1.3 months Median OS 5.83 months
Nivolumab	Blocks PD-1 and PD-L1 interaction [104]	Phase II (NCT02829918)	ORR 11%, DCR 50% Median PFS 3.68 months Median OS 14.24 months
Durvalumab plus GC	PD-L1 inhibitor [70]	Phase III (TOPAZ-1)	OS hazard ratio 0.8 for treatment vs. placebo (*p* < 0.05) PFS hazard ratio 0.75 for treatment vs. placebo (*p* = 0.001)
Pembrolizumab plus GC	PD-1 inhibitor [68]	Phase III (KEYNOTE-966)	Median OS 12.7 months vs. 10.9 months in placebo group (*p* < 0.005) Median PFS 6.5 months vs. 5.6 months in placebo group
Nivolumab plus GC	Blocks PD-1 and PD-L1 interaction [105]	Phase II (BilT-01)	ORR 22.9% Median PFS 6.6 months Median OS 10.6 months

ORR: overall response rate. PFS: progression-free survival. PR: partial response. DCR: disease control rate. OS: overall survival.

**Table 4:** Active or recruiting clinical trials for solid tumors, including cholangiocarcinoma patients.

Trial	Therapy	Condition	Outcome	Status
Phase III ARTEMIDE-Biliary01 NCT06109779	Rilvegostomig + Standard chemotherapy	Locally advanced, unresectable, or metastatic disease at initial diagnosis and any anti-cancer therapy for BTC prior to surgery	The primary endpoint is RFS, and the key secondary endpoint is OS	Recruiting
Phase I/II NCT06439485	Pemigatinib + Atezolizumab + Bevacizumab	Advanced CCA with FGFR2 fusion	Primary endpoint: ORR Secondary endpoint: Safety, RP2D, overall response (complete/partial), PFS, OS	Recruiting
Phase II ComboMATCH NCT05564403	Modified leucovorin, fluorouracil and oxaliplatin (mFOLFOX6) chemotherapy + Binimetinib	BTCs that have spread to other places in the body (advanced) and had progression of cancer after previous treatments (2nd line setting)	Primary endpoint: OS Secondary endpoint: ORR; PFS; DOR, and DCR	Recruiting
Phase Ia/Ib NCT06302621	Pemigatinib + Afatinib	FGFR-altered refractory advanced solid tumors	Primary endpoint: MTD; ORR Secondary endpoint: DCR; DOR; OS, PFS; Best overall response;	Recruiting
Phase I MAST NCT05346484	Monotherapy—CF33-hNIS (hNIS—human sodium iodide symporter) IV or IT Combination—CF33-hNIS pembrolizumab or mFOLFOX	patients with metastatic or advanced solid tumors	Primary endpoint: Frequency and severity of AEs; RP2D. Secondary endpoint: ORR, PFS, OS, DOR, DCR	Recruiting
Phase III NCT06282575	Zanidatamab + Cisplatin + Gemcitabine with or without programmed death protein 1/ligand-1 (PD-1/L1) inhibitor	Advanced HER2-positive BTC	Primary endpoint: PFS; IHC 3+ tumors Secondary endpoints: OS, PFS; confirmed ORR; DOR	Recruiting
Phase I/II NCT05849480	CDX-1140 + Capecitabine + Oxaliplatin + Keytruda	BTC	Primary endpoint: Safe dose; PFS; ORR Secondary endpoint: Safety; 5-yr OS	Recruiting
Phase III FIRST-308 NCT05948475	Tinengotinib vs. Physician’s Choice	(FGFR)-altered, Chemotherapy- and FGFR Inhibitor-Refractory/Relapsed CCA	Primary endpoint: Incidence, duration, and severity of AEs Secondary endpoint: PFS	Recruiting
Phase II BOLSTER NCT05712356	LSTA1 + standard of care (SoC) vs. SoC alone	Previously untreated CCA or those that have progressed after first-line treatment for CCA	Primary endpoint: Incidence of AEs	Active, not recruiting
Phase II FOENIX-CCA4 NCT05727176	Futibatinib	Patients with advanced CCA with FGFR2 fusion or rearrangement	Primary endpoint: ORR by independent central review Secondary endpoint: DOR; PFS; ORR; OS; AEs; QOL	Recruiting
Phase II/III COMPANION-002 NCT05506943	CTX-009 + with Paclitaxel vs. Paclitaxel alone	Patients with previously treated, unresectable advanced or metastatic BTC	Primary endpoint: Best Overall Response Secondary endpoint: PFS; DOR; OS; DCR; Safety; QOL	Active, not recruiting
Phase I NCT04660929	CT-0508 anti-HER2 CAR macrophages	HER2 overexpressing solid tumors	Primary endpoint: Safety & Tolerability Secondary endpoint: ORR; PFS	Active, not recruiting
Phase II HELIX-ICC NCT04251715	mFOLFIRINOX, followed by HAI floxuridine-dexamethasone	Patients with liver-dominant iCCA that cannot be removed by surgery (unresectable)	Primary endpoint: Incidence of abnormal liver function; DCR—during HAI + SYS Secondary endpoint: DCR; PFS; ORR; OS	Active, not recruiting
Phase I/II NCT03907852	TC-210 Gavocabtagene autoleucel (gavo-cel; TC-210) is a novel cell therapy that consists of autologous genetically engineered T cells expressing a single-domain antibody that recognizes human Mesothelin, fused to the CD3-epsilon subunit which, upon expression, is incorporated into the endogenous TCR complex.	Patients with advanced mesothelin-expressing cancers	Primary endpoint: RP2D; efficacy	Active, not recruiting

BTC: Biliary tract cancer; CCA: cholangiocarcinoma; iCCA: intrahepatic cholangiocarcinoma; OS: overall survival; PFS: progression free survival; ORR: objective response rate; DCR: disease control rate; RFS: recurrence free survival; MTD: Maximum tolerated dose; DOR: duration of response; AE: adverse events; RP2D: Recommended Phase 2 Dose; QOL: quality of life; CAR: Chimeric antigen receptor; HAI: hepatic arterial infusion; TCR: T cell receptor.

## Data Availability

No new data were created or analyzed in this study. Data sharing is not applicable to this article.
